# All-Cause Mortality in Patients with Type 2 Diabetes in Association with Achieved Hemoglobin A_1c_, Systolic Blood Pressure, and Low-Density Lipoprotein Cholesterol Levels

**DOI:** 10.1371/journal.pone.0109501

**Published:** 2014-10-27

**Authors:** Hou-Hsien Chiang, Fen-Yu Tseng, Chih-Yuan Wang, Chi-Ling Chen, Yi-Chun Chen, Ting-Ting See, Hua-Fen Chen

**Affiliations:** 1 Section of Endocrinology & Metabolism, Department of Internal Medicine, Far-Eastern Memorial Hospital, New Taipei City, Taiwan; 2 Department of Internal Medicine, National Taiwan University Hospital, College of Medicine, National Taiwan University, Taipei, Taiwan; 3 Graduate Institute of Clinical Medicine, College of Medicine, National Taiwan University, Taipei, Taiwan; Shanghai Institute of Hypertension, China

## Abstract

**Background:**

To identify the ranges of hemoglobin A_1c_ (HbA1c), systolic blood pressure (SBP), and low-density lipoprotein cholesterol (LDL-C) levels which are associated with the lowest all-cause mortality.

**Methods:**

A retrospective cohort of 12,643 type 2 diabetic patients (aged ≥18 years) were generated from 2002 to 2010, in Far-Eastern Memorial Hospital, New Taipei city, Taiwan. Patients were identified to include any outpatient diabetes diagnosis (ICD-9: 250), and drug prescriptions that included any oral hypoglycemic agents or insulin prescribed during the 6 months following their first outpatient visit for diabetes. HbA1c, SBP, and LDL-C levels were assessed by the mean value of all available data, from index date to death or censor date. Deaths were ascertained by matching patient records with the Taiwan National Register of Deaths.

**Results:**

Our results showed general U-shaped associations, where the lowest hazard ratios occurred at HbA1c 7.0–8.0%, SBP 130–140 mmHg, and LDL-C 100–130 mg/dL. The risk of mortality gradually increases if the patient's mean HbA1c, SBP, or LDL-C during the follow-up period was higher or lower than these ranges. In comparison to the whole population, the adjusted hazard ratio (95% CI) for patients with HbA1c 7.0–8.0%, SBP 130–140 mmHg, and LDL-C 100–130 mg/dL were 0.69 (0.62–0.77), 0.80 (0.72–0.90), and 0.68 (0.61–0.75), respectively.

**Conclusions:**

In our type 2 diabetic cohort, the patients with HbA1c 7.0–8.0%, SBP 130–140 mmHg, or LDL-C 100–130 mg/dL had the lowest all-cause mortality. Additional research is needed to confirm these associations and to further investigate their detailed mechanisms.

## Introduction

The randomized controlled trials to understand the benefits of very low hemoglobin A_1c_ (HbA1c; <6.0–6.5%) levels for type 2 diabetes patients in improving survival rates and reducing macrovascular complications had provoked much controversy in the medical field. A ten-year follow-up study performed by the United Kingdom Prospective Diabetes Study (UKPDS) Group demonstrated that long-term cardiovascular protection can be achieved by early intensive glycemic control [1], [2]. However, the Action to Control Cardiovascular Risk in Diabetes (ACCORD) Trial, the Action in Diabetes and Vascular Disease Trial (ADVANCE), and the Veterans Affairs Diabetes Trial (VADT) failed to show definite reductions of cardiovascular events and overall mortalities among the patients who were receiving intensive glycemic controls [3]–[6]. Furthermore, the ACCORD trial was terminated prematurely due to an increased mortality rate for the patient group targeting HbA1c <6.0% [3].

The controversy regarding the relation between achieved HbA1c and survival rates is also present among observational studies. Currie et al. reported from the UK General Practice Research Database (GPRD) that low and high HbA1c were associated with increased all-cause mortality and cardiovascular events [7]. The U-shaped relationship observed in this study was similar to those reported in other retrospective studies [8], [9]. However, some other studies have shown that type 2 diabetic patients with the lowest HbA1c exhibited the lowest all-cause mortality [10], [11].

Cardiovascular risk in patients with diabetes has been shown to be graded and continuous across the range of systolic blood pressure levels [12]–[14]. Clinical trials have demonstrated the reduction of cardiovascular events and nephropathy by lowering diabetic patients' blood pressures to 140 mmHg systolic and 80 mmHg diastolic [15]–[17]. To further reduce vascular complications for diabetic patients, clinical guidelines recommend maintaining systolic blood pressures of less than 130 mmHg [18], [19]. However, evidence supporting this commonly recommended blood pressure goal was not rigorously established. Furthermore, the ACCORD trial showed that for the type 2 diabetic patients who are at high risks of cardiovascular events, targeting a systolic blood pressure of <120 mmHg, as compared with <140 mmHg, did not significantly change the occurrence rates of cardiovascular events or all-cause mortality [20].

An increased prevalence of lipid abnormalities is observed in patients with type 2 diabetes. Randomized controlled trials of statin therapy demonstrated significant primary and secondary prevention of cardiovascular events in diabetic patients [21]–[25]. Meta-analyses of 14 randomized trials of statin therapy demonstrated a 21% proportional reduction in major vascular events and a 9% reduction in all-cause mortality, for each mmol/L reduction in low-density lipoprotein cholesterol (LDL-C) [26]. However, in most of the above trials the end-of-treatment LDL-C of the statin group was more than 100 mg/dL. In the Treating to New Targets (TNT) study, patients with diabetes, stable coronary artery disease, and LDL-C of <130 mg/dL, were randomized to receive atorvastatin 10 or 80 mg per day [27]. The achieved LDL-C levels for the patients who received either 10 and 80 mg of atorvastatin per day were 98.6 and 77.0 mg/dL, respectively. Compared to the low-dosage group, the high-dosage group had a 25% proportional reduction in major cardiovascular events, but there was no significant difference observed for all-cause mortality.

Prior large randomized controlled trials and observational studies did not show concurrent results regarding the benefits of survival rate, when HbA1c level was intensively controlled in patients with type 2 diabetes mellitus. Furthermore, very few observational studies was performed to understand the association between mortality and systolic blood pressure or LDL-C levels in these diabetic patients. To improve the treatment of type 2 diabetes, we aimed to identify the ranges of HbA1c, systolic blood pressure, and LDL-C levels associated with the lowest all-cause mortality, and to further understand if the risk of all-cause mortality is increased when these metabolic factors are extremely low.

## Methods

### Data collection

This retrospective cohort study was conducted in Far-Eastern Memorial Hospital, which is the largest general hospital in New Taipei City among the medical centers in Taiwan. The data was obtained from the computerized medical database from our hospital, which includes demographic information, medical history, laboratory test results, and drug prescriptions. Medical history was coded according to the International Classification of Diseases, Ninth Revision (ICD-9) in the outpatient and inpatient database (maximum of five leading discharge diagnoses). By 1996, the Taiwan National Health Insurance (NHI) program had covered most of the population (99%) in Taiwan. These diagnoses were transferred to the Taiwan Bureau of National Health Institute (BNHI) for payments. To ensure the precision and accuracy of claim data, BNHI performs expert reviews with random samplings of every 50–100 outpatient and inpatient claims from each hospital, quarterly. Falsification of diagnosis reports will result in severe penalties from the BNHI [28], [29].

### Patients and setting

Patients were indentified to include any outpatient diabetes diagnosis (ICD-9: 250), and drug prescriptions that included any oral hypoglycemic agents or insulin prescribed during the 6 months following their first outpatient visit for diabetes. The patients who did not have at least 12 months of follow-up after their respective index date, which is defined as the date half year after the first outpatient visit for diabetes, were excluded from our study [7]. The baseline period was defined as the 6 months between the first outpatient visit for diabetes and the index date. We further excluded those patients who were under 18 years old at index date, and type 1 diabetic patients. Type 1 diabetes mellitus was identified by ICD-9: (250.x1 or 250.x3) and catastrophic illness registration cards. In Taiwan, BNHI issues catastrophic illness registration cards to patients who were diagnosed with mayor illnesses such as type 1 diabetes. These patients are exempt from copayment to the NHI if they seek medical care for their associating illnesses. The final diabetic cohort consisted a total of 17,837 patients.

Patients were followed up from their respective index date until the occurrence of all-cause death. If no death was recorded, the date of censoring was defined as the date of study termination (December 31, 2010). The study protocol was approved by The Research Ethics Committee of Far-Eastern Memorial Hospital, and waived the need for informed consent; protocol number 100073-F.

### Outcome

The outcome measure was all-cause mortality, which was ascertained by matching the computerized data file of the Taiwan National Register of Deaths with that patients' unique personal identification numbers (PIN). All data were obtained between January 1, 2002, to December 31, 2010, inclusively. Time of death was given in years and months, and we further artificially assigned the last day of the month as the day of death. After the correspondence of the medical and mortality database with PIN, records were anonymized.

### Assessment of achieved HbA1c, blood pressure, and LDL-C levels

HbA1c was measured in whole blood using ion exchange high-performance liquid chromatography (G7 Analyzer, Tosoh Bioscience, Tokyo, Japan). LDL-C was analyzed using a biochemistry automatic analyzer (7600 Clinical Analyzer, Hitachi High-Technologies Corporation, Tokyo, Japan). To explore the risk of mortality associated with HbA1c, we categorized HbA1c in 1.0% segments, resulting in 6 groups (from <6.0% to ≥10%). LDL-C was categorized as <70, 70–100, 100–130, 130–160, and ≥160 mg/dL. Blood pressure was measured with aneroid sphygmomanometer while patients were in a seated position at each outpatient visit, and written into the electronic medical record by physicians. Systolic blood pressure (SBP) was recorded by research assistants as one random blood pressure value per year. SBP was categorized in 10 mmHg segments, resulting in 6 groups (from <120 mmHg to ≥160 mmHg).

### Assessment of covariates

Covariates evaluated in this analysis were age at index date, sex, pre-existing comorbidities, and baseline use of insulin. Pre-existing major comorbidities of type 2 diabetic patients can include prior myocardial infarction (ICD-9: 410, 412), congestive heart failure (ICD-9: 402.01, 402.11, 402.91, 404.01, 404.03, 404.11, 404.13, 404.91, 404.93 and 428), prior stroke (ICD-9: 430–434, 436), malignant neoplasm (ICD-9: 140–208), and chronic kidney disease (ICD-9: 250.4, 274.1, 283.11, 403.1, 404.2, 404.3, 440.1, 442.1, 447.3, 572.3, 580–588, 642.1, and 646.2) [30]–[34]. These comorbidities were identified by the diagnosis codes in the patient records, occurring at least once before index date in either the outpatient or inpatient database.

### Statistical analysis

Patient clinical characteristics during the baseline period are reported as mean ± standard deviation or number (percentage). Cox proportional hazards regression analyses (adjusted for potential confounders) were used to estimate the hazard ratio of each categorized group for all-cause mortality relative to the population mean. The adjusted Cox models were calculated with the non-stepwise method. The P values were two-sided, and values of less than 0.05 were considered statistically significant. All statistical analyses were performed with SPSS version 17.0 (SPSS Inc., Chicago, IL, USA).

Patients with missing HbA1c, systolic blood pressure, or lipid levels were excluded in the analyses. Therefore, 29.1% of the patients with at least one absent value of HbA1c, SBP, and LDL-C were excluded. A total of 12,643 patients were included in the analyses. We introduced the achieved HbA1c, SBP, and LDL-C as time-fixed covariates, calculated as the mean of all observations recorded between the index date and the respective death or censoring date. To address the dynamic nature of HbA1c, SBP and LDL-C over time, we performed sensitivity analyses introducing HbA1c, SBP and LDL-C into the Cox model in an updated, cumulative, yearly mean value (with the last observation carried forward for missing data).

To determine the relationship between HbA1c and mortality, subgroup analysis was performed with patients who were given insulin (with or without oral hypoglycemic agents) or only oral hypoglycemic agents during the baseline period. To determine the relationship between SBP or LDL-C, and mortality, subgroup analysis was performed with patients who had associated baseline diagnoses and drug prescriptions (anti-hypertensive drugs include angiotensin converting enzyme inhibitor, angiotensin receptor blocker, β-blocker, calcium channel blocker, diuretic, α-blocker, hydralazine, and methyldopa; lipid-lowering drugs include statin, ezetimibe, fibrate, and cholestyramine), and patients who did not.

## Results

### Clinical Characteristics

Out of the 12,643 type 2 diabetic patients, there were 1,278 deaths that occurred at a mean follow-up duration of 5.6±2.4 years. This follow-up is equivalent to 70,902 person-years. The mean patient age was 57.2±12.2 years, and 50.4% were male (Table 1). The mean HbA1c for these patients, from index date to death or censor date, was 7.99%±1.50%. The mean systolic blood pressure was 135.5±11.6 mmHg and the mean plasma LDL-C was 110.0±27.0 mg/dL. The most commonly prescribed anti-diabetic drugs during the baseline period were sulfonylurea (76.7%) and metformin (75.7%), followed by insulin (15.3%) and thiazolidinedione (14.0%).

**Table 1:** Clinical characteristics of the 12,643 patients with type 2 diabetes mellitus.

Characteristics	
Age (year)*	57.2±12.2
Male	6368 (50.4%)
HbA1c (%)†	7.99±1.50
SBP (mmHg)†	135.5±11.6
LDL-C (mg/dL)†	110.0±27.0
Baseline medications:	
Insulin	1938 (15.3%)
Metformin	9566 (75.7%)
Sulfonylurea	9699 (76.7%)
Meglitinide	1103 (8.7%)
Thiazolidinedione	1774 (14.0%)
α-Glucosidase inhibitor	927 (7.3%)
DPP-4 inhibitor	81 (0.6%)
β-blocker	2210 (17.5%)
Calcium channel blocker	3046 (24.1%)
ACE inhibitor or ARB	4897 (38.7%)
Diuretic	1809 (14.3%)
Antiplatelet	3549 (28.1%)
Statin	2211 (17.5%)
Baseline comorbidities:	
Prior myocardial infarction	1677 (13.3%)
Congestive heart failure	588 (4.7%)
Prior stroke	1157 (9.2%)
Malignant neoplasm	287 (2.3%)
Chronic kidney disease	2076 (16.4%)

Data are n (%) or mean ± standard deviation. HbA1c: hemoglobin A_1c_; SBP: systolic blood pressure; LDL-C: low-density lipoprotein cholesterol; DPP-4: dipeptidyl peptidase-4; ACE: angiotensin converting enzyme; ARB: angiotensin receptor blocker.

*Age at index date was used for calculation.

† Mean HbA1c, SBP, and LDL-C were the mean of any values recorded between the index date and death or censor.

### All-cause mortality and achieved HbA1c, SBP, and LDL-C levels

**Figure 1:**
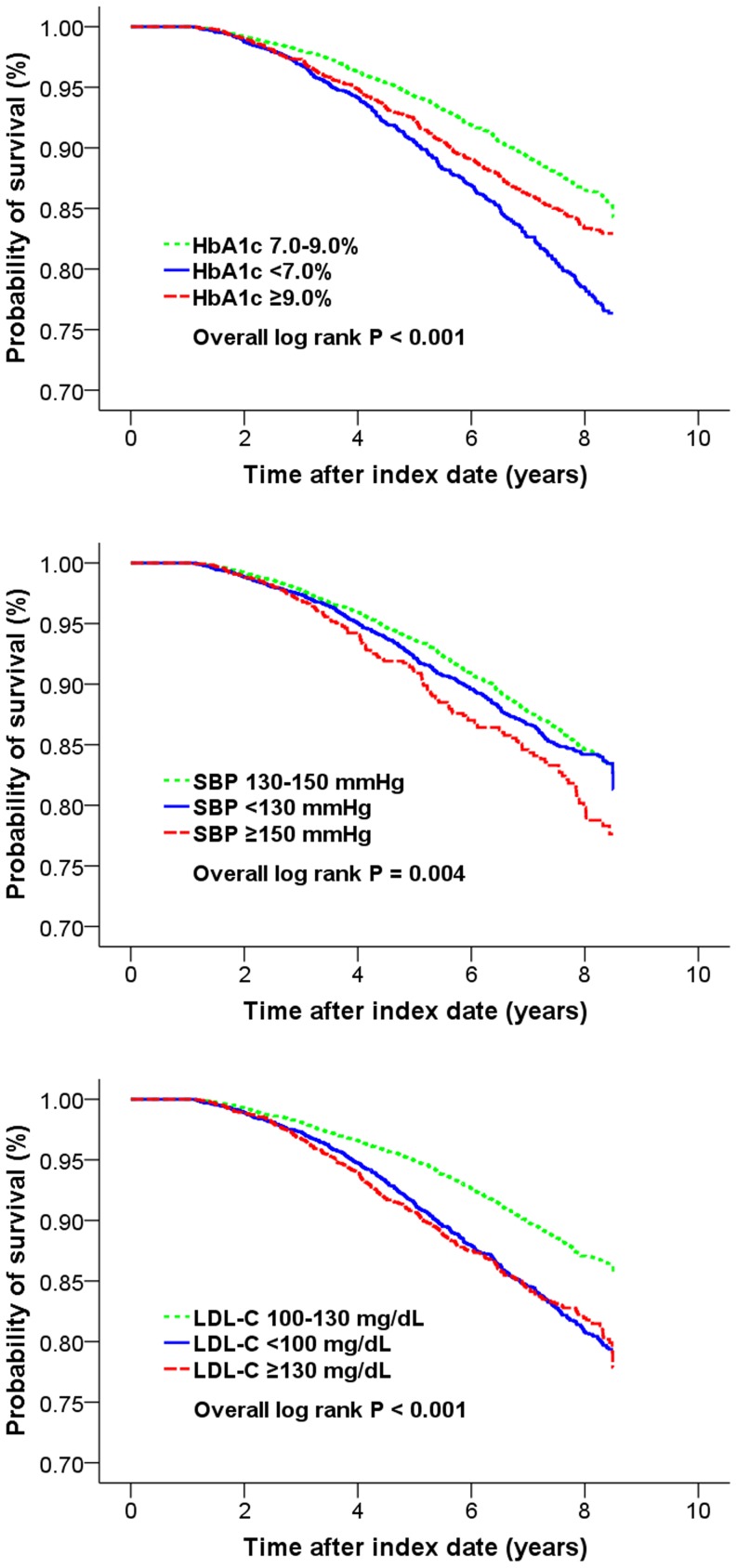
Kaplan-Meier survival curves according to post-index mean HbA1c, SBP, and LDL-C levels.

In the univariable analyses, all-cause mortality was related to HbA1c, SBP and LDL-C in general U-shaped patterns. Kaplan-Meier survival curves in Figure 1 showed the unadjusted relative risks of mortality, according to high, usual, or low HbA1c, SBP, and LDL-C levels (with reference groups of HbA1c 7.0–9.0%, SBP 130–150 mmHg, and LDL-C 100–130 mg/dL).

**Figure 2:**
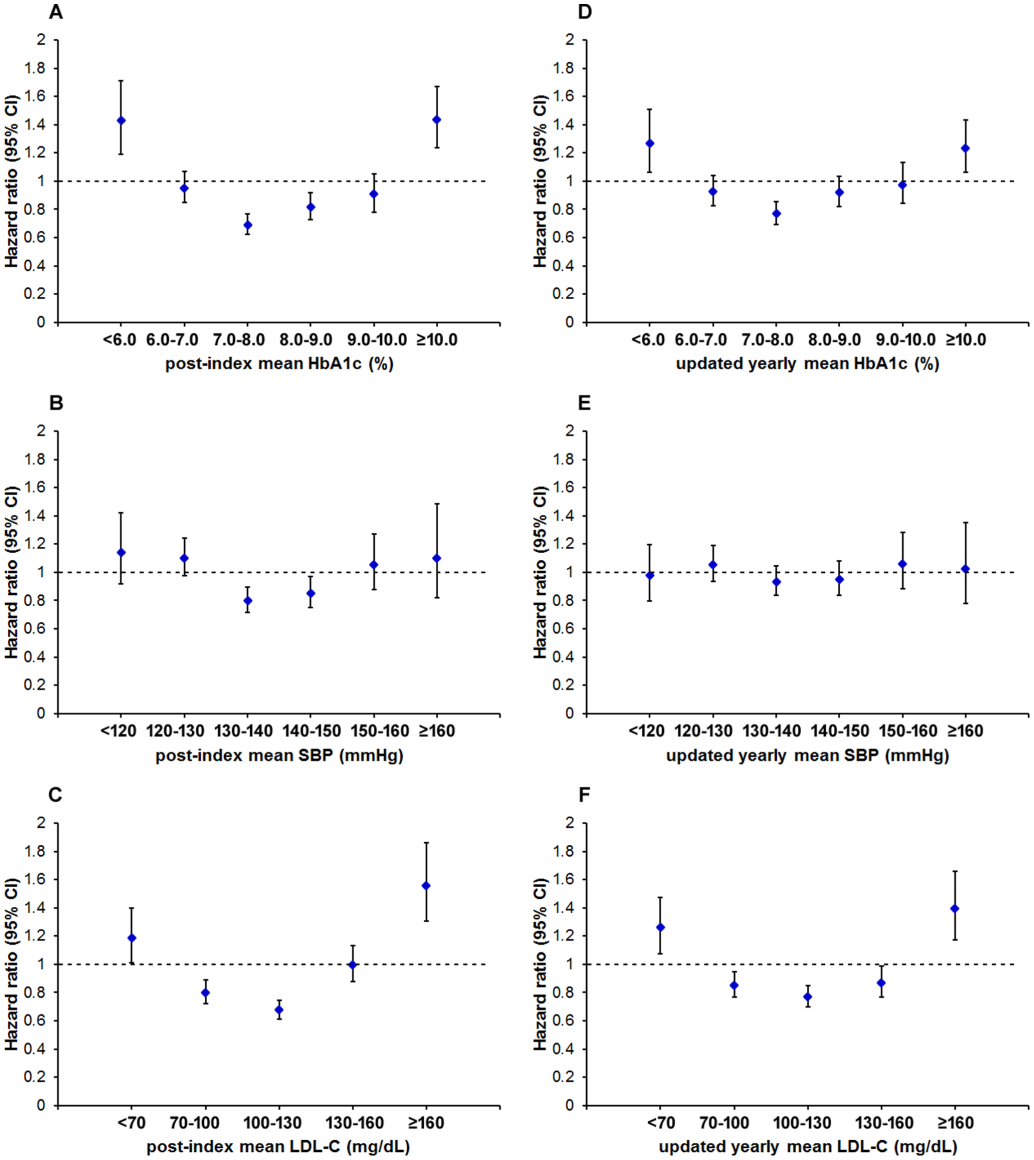
Adjusted hazard ratios for all-cause mortality according to time-fixed or time-dependent HbA1c, SBP and LDL-C levels. Cox proportional hazards regression analyses were used to calculate hazard ratios relative to the population mean. Potential confounders were adjusted as Model 2 in Table 2, and Model 2,4,6 in Table 3. Vertical error bars indicate 95% confidence intervals.

In the multivariable analyses with adjustment for potential confounders, HbA1c category 7.0–8.0% had the lowest hazard ratio for all-cause mortality (Table 2). The risk of mortality was shown to gradually increase, if the patient's mean HbA1c during follow-up period was higher or lower than the range between 7.0–8.0%. In comparison to the whole population, the adjusted hazard ratio (95% CI) for patients with HbA1c 7.0–8.0% was 0.69 (0.62–0.77) (Model 2 in Table 2, also see Figure 2).

**Table 2:** Cox proportional hazard models for all-cause mortality introducing achieved HbA1c, SBP, and LDL-C as post-index mean values.

	Patient number	Mortality rate (per 1000 person-years)	Model 1		Model 2	
			Hazard ratio (95% CI)	*P* value	Hazard ratio (95% CI)	*P* value
HbA1c (%)*						
<6.0	444	41.7	1.37 (1.14–1.64)	<0.001	1.43 (1.19–1.71)	<0.001
6.0–7.0	2665	20.9	0.88 (0.79–0.99)	0.034	0.95 (0.85–1.07)	0.4
7.0–8.0	4187	14.3	0.68 (0.61–0.75)	<0.001	0.69 (0.62–0.77)	<0.001
8.0–9.0	2758	18.0	0.83 (0.74–0.93)	0.002	0.82 (0.73–0.92)	0.001
9.0–10.0	1408	17.2	0.98 (0.84–1.13)	0.8	0.91 (0.78–1.05)	0.20
≥10.0	1181	22.3	1.50 (1.30–1.74)	<0.001	1.44 (1.24–1.67)	<0.001
SBP (mmHg)*						
<120	834	15.9	1.06 (0.86–1.32)	0.6	1.14 (0.92–1.42)	0.23
120–130	3182	18.9	1.06 (0.94–1.20)	0.33	1.10 (0.98–1.25)	0.12
130–140	4617	16.6	0.66 (0.60–0.72)	<0.001	0.80 (0.72–0.90)	<0.001
140–150	2692	18.8	0.84 (0.74–0.96)	0.009	0.85 (0.75–0.97)	0.014
150–160	941	21.6	1.14 (0.95–1.36)	0.17	1.06 (0.88–1.27)	0.6
≥160	377	20.1	1.15 (0.85–1.54)	0.4	1.10 (0.82–1.48)	0.5
LDL-C (mg/dL)*						
<70	676	33.7	1.30 (1.11–1.53)	0.001	1.19 (1.01–1.40)	0.038
70–100	3604	19.6	0.80 (0.72–0.89)	<0.001	0.80 (0.72–0.89)	<0.001
100–130	6051	14.1	0.81 (0.72–0.90)	<0.001	0.68 (0.61–0.75)	<0.001
130–160	1806	19.5	0.94 (0.82–1.06)	0.31	1.00 (0.87–1.13)	0.9
≥160	506	31.9	1.56 (1.30–1.86)	<0.001	1.56 (1.30–1.86)	<0.001

The models used Cox proportional hazards regression analyses adjusted for potential confounders. The hazard ratios relative to the population mean were calculated.

*HbA1c, SBP, and LDL-C were calculated as the mean of any values recorded between the index date and death or censor.

Model 1 adjusted for age and sex.

Model 2 included the confounders in model 1, plus pre-existing myocardial infarction, congestive heart failure, stroke, malignant neoplasm, chronic kidney disease, use of insulin, any anti-hypertensive drug, any lipid-lowering drug, and antiplatelet.

The relationships between all-cause mortality and SBP or LDL-C were similar to that between all-cause mortality and HbA1c. In the multivariable analyses adjusted for potential confounders, the lowest hazard ratio for mortality occurred at SBP 130–140 mmHg and LDL-C 100–130 mmHg (Table 2). In comparison to the whole population, the adjusted hazard ratio (95% CI) for patients with SBP 130–140 mmHg and LDL-C 100–130 mg/dL were 0.80 (0.72–0.90), and 0.68 (0.61–0.75), respectively (Model 2 in Table 2, also see Figure 2). Since the patients with HbA1c 7.0–8.0%, SBP 130–140 mmHg, or LDL 100–130 mg/dL have the lowest mortality, we calculated all the hazard ratios again by using these respective groups as the references. The results were shown in Table S1, Table S2, and Table S3.

In the sensitivity analyses using updated yearly mean, the overall U-shaped relationships between mortality and HbA1c or LDL-C preserved, although the strengths of the associations weakened (Table 3). In comparison to the whole population, the adjusted hazard ratio (95% CI) for patients with HbA1c 7.0–8.0% and LDL-C 100–130 mg/dL were 0.77 (0.69–0.85), and 0.77 (0.70–0.85), respectively (Model 2,6 in Table 3, also see Figure 2). However, the association between mortality and updated yearly mean SBP was not obvious.

**Table 3:** Cox proportional hazard models for all-cause mortality introducing achieved HbA1c, SBP, or LDL-C as updated, cumulative, yearly mean values.

	Hazard ratio (95% CI)	*P* value	Hazard ratio (95% CI)	*P* value
HbA1c (%)*	Model 1		Model 2	
<6.0	1.21 (1.02–1.44)	0.033	1.27 (1.06–1.51)	0.008
6.0–7.0	0.86 (0.77–0.97)	0.011	0.93 (0.83–1.04)	0.21
7.0–8.0	0.76 (0.68–0.84)	<0.001	0.77 (0.69–0.85)	<0.001
8.0–9.0	0.94 (0.83–1.05)	0.25	0.92 (0.82–1.03)	0.16
9.0–10.0	1.04 (0.90–1.21)	0.6	0.98 (0.84–1.13)	0.7
≥10.0	1.30 (1.12–1.50)	0.001	1.23 (1.06–1.43)	0.006
SBP (mmHg)*	Model 3		Model 4	
<120	0.92 (0.76–1.13)	0.4	0.98 (0.80–1.20)	0.8
120–130	1.02 (0.90–1.15)	0.8	1.06 (0.94–1.19)	0.4
130–140	0.96 (0.86–1.07)	0.4	0.93 (0.84–1.04)	0.23
140–150	0.98 (0.86–1.11)	0.7	0.95 (0.84–1.08)	0.4
150–160	1.10 (0.91–1.32)	0.32	1.06 (0.88–1.28)	0.5
≥160	1.04 (0.79–1.36)	0.8	1.03 (0.78–1.35)	0.9
LDL-C (mg/dL)*	Model 5		Model 6	
<70	1.33 (1.14–1.56)	<0.001	1.26 (1.08–1.47)	0.004
70–100	0.87 (0.78–0.97)	0.009	0.85 (0.77–0.94)	0.002
100–130	0.75 (0.68–0.83)	<0.001	0.77 (0.70–0.85)	<0.001
130–160	0.84 (0.74–0.95)	0.007	0.87 (0.77–0.99)	0.034
≥160	1.37 (1.15–1.62)	<0.001	1.39 (1.17–1.66)	<0.001

The models used Cox proportional hazards regression analyses adjusted for potential confounders. The hazard ratios relative to the population mean were calculated.

*HbA1c, SBP, or LDL-C was introduced in an updated, cumulative, yearly mean value (with the last observation carried forward for missing data).

Model 1 adjusted for age, sex, mean SBP, and LDL-C.

Model 3 adjusted for age, sex, mean HbA1c, and LDL-C.

Model 5 adjusted for age, sex, mean HbA1c, and SBP.

Model 2,4,6 included the confounders in model 1,2,3, respectively, plus pre-existing myocardial infarction, congestive heart failure, stroke, malignant neoplasm, chronic kidney disease, use of insulin, any anti-hypertensive drug, any lipid-lowering drug, and antiplatelet.

### Subgroup analyses

For the patients with baseline use of insulin, the lowest hazard ratio for mortality occurred at HbA1c 8.0–9.0% with adjusted hazard ratio (95% CI) 0.73 (0.58–0.91), in comparison to the whole insulin subgroup (Model 1 in Table 4). For the patients with baseline hypertension, the U-shaped relation between mortality and SBP was preserved (Model 3 in Table 4). However, for patients with baseline hyperlipidemia, the U-shaped relation between mortality and LDL-C was less prominent than those without baseline hyperlipidemia (Model 5,6 in Table 4).

**Table 4:** Cox proportional hazard models in subgroups.

	Patient number	Mortality rate (per 1000 person-years)	Hazard ratio (95% CI)	*P* value	Patient number	Mortality rate (per 1000 person-years)	Hazard ratio (95% CI)	*P* value
HbA1c (%)*			Model 1: with insulin				Model 2: with only oral hypoglycemic agents	
<6.0	53	103.6	1.80 (1.24–2.61)	0.002	391	35.0	1.35 (1.09–1.66)	0.005
6.0–7.0	247	44.1	1.07 (0.82–1.39)	0.6	2418	18.8	0.91 (0.80–1.04)	0.16
7.0–8.0	502	34.6	0.80 (0.65–0.98)	0.026	3685	11.8	0.64 (0.57–0.73)	<0.001
8.0–9.0	477	29.1	0.73 (0.58–0.91)	0.005	2281	13.9	0.85 (0.74–0.97)	0.019
9.0–10.0	339	26.7	0.77 (0.59–1.00)	0.047	1069	14.4	1.00 (0.83–1.19)	1.0
≥10.0	320	33.5	1.17 (0.90–1.51)	0.24	861	18.4	1.51 (1.25–1.81)	<0.001
SBP (mmHg)*			Model 3: with baseline hypertension				Model 4: without baseline hypertension	
<120	238	27.9	1.16 (0.85–1.58)	0.35	596	11.5	1.14 (0.82–1.59)	0.4
120–130	1177	27.9	1.16 (0.99–1.35)	0.071	2005	13.9	1.04 (0.84–1.30)	0.7
130–140	2441	20.5	0.78 (0.68–0.90)	<0.001	2176	12.2	0.84 (0.67–1.04)	0.11
140–150	1295	21.6	0.86 (0.74–1.00)	0.050	897	13.1	0.80 (0.61–1.05)	0.11
150–160	698	23.7	1.04 (0.85–1.28)	0.7	243	15.2	1.09 (0.71–1.69)	0.7
≥160	300	20.4	1.06 (0.76–1.48)	0.7	77	19.3	1.15 (0.57–2.33)	0.7
LDL-C (mg/dL)*			Model 5: with baseline hyperlipidemia				Model 6: without baseline hyperlipidemia	
<70	154	24.4	1.03 (0.66–1.61)	0.9	522	35.8	1.22 (1.02–1.45)	0.029
70–100	787	18.5	0.96 (0.75–1.24)	0.8	2817	19.8	0.77 (0.69–0.86)	<0.001
100–130	1293	13.9	0.79 (0.62–0.99)	0.041	4758	14.1	0.65 (0.58–0.73)	<0.001
130–160	487	15.2	0.92 (0.68–1.26)	0.6	1319	20.8	1.01 (0.87–1.16)	0.9
≥160	166	30.8	1.39 (0.98–1.98)	0.061	340	32.4	1.63 (1.32–2.01)	<0.001

The models used Cox proportional hazards regression analyses adjusted for potential confounders. The hazard ratios relative to the population mean were calculated.

*HbA1c, SBP, and LDL-C were calculated as the mean of any values recorded between the index date and death or censor.

Model 1,2 adjusted for age, sex, mean SBP, LDL-C, pre-existing myocardial infarction, congestive heart failure, stroke, malignant neoplasm, chronic kidney disease, use of insulin, any anti-hypertensive drug, any lipid-lowering drug, and antiplatelet.

Model 3,4 included the confounders in model 1,2 minus mean SBP, but plus mean HbA1c.

Model 5,6 included the confounders in model 1,2 minus mean LDL-C, but plus mean HbA1c.

## Discussion

Our study revealed the ranges of HbA1c, SBP, and LDL-C that were associated with the lowest all-cause mortality for the type 2 diabetic cohort. Increases or decreases from these references were associated with higher mortality, which exhibited general U-shaped relations. Our study showed significantly elevated risks of all-cause mortality at extremely low HbA1c and LDL-C, while no elevated risk of mortality was seen at extremely low SBP in comparison to the whole population.

In the sensitivity analyses using updated yearly mean with last observation carried forward, the overall U-shaped relationships preserved for HbA1c and LDL-C, but the association between mortality and SBP was not obvious. One possible reason is that in our study, SBP was recorded as only one random blood pressure value per year, and therefore, the random value is too fluctuant to reflect a patient's condition.

### HbA1c and mortality

The U-shaped association between HbA1c and mortality found in this study was comparable with some of the prior randomized controlled trials and observational studies [3], [6]–[9]. Compared to ACCORD, ADVANCE, and VADT trials, in which high-risk type (old age, long duration of diabetes, and high cardiovascular risk) patients were enrolled, our study has a more comprehensive population that included all type 2 diabetic patients aged 18 years and older.

However, it is difficult to determine whether the elevated risk of mortality at low HbA1c levels is an effect of intensive glucose control, or a result of some vulnerable factors associated with low HbA1c, or both. To mitigating the confounding effects of low HbA1c-associated factors, we adjusted five baseline comorbidities, including prior myocardial infarction, congestive heart failure, prior stroke, malignant neoplasm, and chronic kidney disease. These comorbidities were included because cardiovascular disease and malignant neoplasm are the two leading causes of death for patients with type 2 diabetes [35]. In addition, diabetic nephropathy and other causes of renal diseases are common complications of diabetes that are associated with mortality [36]. However, there are still other vulnerable factors, and many of which are difficult to quantify, for instance, nutritional status and frailty. These potential confounders is the weakness of observational studies. Hypoglycemia is the most accepted mechanism of increased mortality related to intensive glycemic control. In randomized controlled trials, the occurrence of hypoglycemia was associated with increased risks of a range of adverse clinical outcomes and mortality [37], [38]. Possible mechanisms by which hypoglycemia might cause cardiovascular disease or death include sympathoadrenal activation, abnormal cardiac repolarization, increased thrombogenesis, vasoconstriction, and the release of inflammatory mediators and cytokines [39].

### SBP and mortality

Our study demonstrated a U-shaped association between post-index mean SBP and mortality, which is compatible with some prior studies. In the International Verapamil SR-Trandolapril Study (INVEST), the post-hoc analysis of participants who were at least 50 years old and had diabetes and coronary artery disease indicated that the all-cause mortality rate was 11.0% in the tight-control group (average SBP<130 mmHg) versus 10.2% in the usual-control group (average SBP 130–139 mmHg) (adjusted hazard ratio, 1.20; 95% CI, 0.99–1.45) [40]. When extended follow-up was included, risk of all-cause mortality was 22.8% and 21.8% in the tight control and the usual control group, respectively (adjusted hazard ratio, 1.15; 95% CI, 1.01–1.32). Vamos et al. reported from UK General Practice Research Database (GPRD) that for adult patients with a new diagnosis of type 2 diabetes, low blood pressure achieved in the first year of treatment was associated with an increased risk of all-cause mortality [41]. However, compared with SBP 130–139 mmHg, the adjusted RR begins to increase significantly when SBP is lower than 120 mmHg. Recently, the Eighth Joint National Committee (JNC 8) guideline has raised the SBP goal in diabetic patients to 140 mmHg, and the results of our study are compatible with this change [42].

### LDL-C and mortality

Our study did not show a reduction of mortality at extremely low LDL-C levels, even for patients who had baseline diagnoses of hyperlipidemia and lipid-lowering drugs. However, benefits of statin are supposed to be the greatest in people with high baseline cardiovascular risk. Clinical trials in patients with high cardiovascular risk, such as those with acute coronary syndromes or previous cardiovascular events, have demonstrated that high doses of statins to achieve an LDL-C of 70 mg/dL led to a significant reduction in further events, and a trend toward reduction of all-cause mortality [43], [44]. In comparison, our subgroup with diabetes and hyperlipidemia with has low cardiovascular risk, and thus, get less benefits from aggressive LDL-C reduction. With regard our whole diabetic cohort, the elevated mortality with low LDL-C level may reflect frailty or subclinical diseases associated with low LDL-C levels. Some observational studies indicated that low plasma LDL-C is associated with higher all-cause mortality in older patients [45], [46]. However, our study is the first observational study, in our best knowledge, to investigate the relation between LDL cholesterol and all-cause mortality in people with type 2 diabetes. Further studies are required to confirm our findings.

### Study limitations

There are several limitations in this study. First of all, our study was not randomized. We adjusted some recognized confounding factors, but unmeasured confounding might still explain a certain portion of the results. In our study, patients' smoking history, body mass index, and family history of major systemic diseases were not complete. Secondly, some patient data contained missing laboratory tests and blood pressure data. These absences of data is mainly caused by some physicians who did not check or record them as per protocol. Among the missing data, LDL-C had the highest rate of absence (27.8%). Variability in the frequency of HbA1c, SBP, and LDL-C measurement might have introduced bias. However, we analyzed the Cox models by two methods (time-fixed mean of all observations, and time-dependent updated, cumulative, yearly mean) and the results were similar. Furthermore, we solely used the ICD-9 diagnosis codes to identify baseline comorbidities, while coding imperfection might have occurred. Also, we could not get the data of duration of diabetes, so we used baseline use of insulin as a covariate in the Cox regression analyses instead. Finally, we did not investigate the causes of deaths, which might provide more information about the relations between mortality and achieved HbA1c, blood pressure, and lipid levels. In the Taiwan National Register of Deaths, specific causes of death were classified into categories according to ICD-9 (2002–2007) and ICD-10 (2008–2009) codes [28]. In our study population, 30.8% of death was classified into diabetes (ICD-9: 250, ICD-10: E10-E14) as its primary cause. The classification system is not precise enough for us to perform further investigations.

## Conclusions

In our type 2 diabetic cohort, the patients with HbA1c 7.0–8.0%, SBP 130–140 mmHg, or LDL-C 100–130 mg/dL had the lowest all-cause mortality. The risk of mortality was significantly increased when HbA1c and LDL-C were extremely low. Additional research is needed to confirm these associations and to investigate their detailed mechanisms.

## Supporting Information

Table S1 **Cox proportional hazard models for all-cause mortality introducing achieved HbA1c, SBP, and LDL-C as post-index mean values.**
(DOCX)

Table S2 **Cox proportional hazard models for all-cause mortality introducing achieved HbA1c, SBP, or LDL-C as updated, cumulative, yearly mean values.**
(DOCX)

Table S3 **Cox proportional hazard models in subgroups.**
(DOCX)
