# Supplementary material for: All-Cause Mortality in Patients with Type 2 Diabetes in Association with Achieved Hemoglobin A1c, Systolic Blood Pressure, and Low-Density Lipoprotein Cholesterol Levels
Source: PLoS One. 2014 Oct 27;9(10):e109501. doi: 10.1371/journal.pone.0109501 (PMC4210124; doi:10.1371/journal.pone.0109501)
Supplement: Table S1 — Cox proportional hazard models for all-cause mortality introducing achieved HbA1c, SBP, and LDL-C as post-index mean values. (DOCX) [file pone.0109501.s001.docx]

**Table S1. Cox proportional hazard models for all-cause mortality introducing achieved HbA1c, SBP, and LDL-C as post-index mean values**

|  | Patient number | Mortality rate (per 1000 person-years) | Model 1 | | Model 2 | |
| --- | --- | --- | --- | --- | --- | --- |
|  |  |  | Hazard ratio (95% CI) | *P* value | Hazard ratio (95% CI) | *P* value |
| HbA1c (%)* | | | | | | |
| <6.0 | 444 | 41.7 | 2.03 (1.61-2.56) | <0.001 | 2.06 (1.64-2.61) | <0.001 |
| 6.0-7.0 | 2665 | 20.9 | 1.31 (1.12-1.53) | <0.001 | 1.38 (1.17-1.61) | <0.001 |
| 7.0-8.0 | 4187 | 14.3 | reference |  | reference |  |
| 8.0-9.0 | 2758 | 18.0 | 1.23 (1.05-1.45) | 0.011 | 1.18 (1.00-1.39) | 0.045 |
| 9.0-10.0 | 1408 | 17.2 | 1.45 (1.19-1.76) | <0.001 | 1.31 (1.08-1.60) | 0.007 |
| ≥10.0 | 1181 | 22.3 | 2.23 (1.83-2.71) | <0.001 | 2.08 (1.70-2.53) | <0.001 |
| SBP (mmHg)* | | | | | | |
| <120 | 834 | 15.9 | 1.32 (1.02-1.71) | 0.037 | 1.43 (1.10-1.85) | 0.008 |
| 120-130 | 3182 | 18.9 | 1.32 (1.15-1.52) | <0.001 | 1.38 (1.19-1.58) | <0.001 |
| 130-140 | 4617 | 16.6 | reference |  | reference |  |
| 140-150 | 2692 | 18.8 | 1.05 (0.90-1.21) | 0.6 | 1.06 (0.92-1.23) | 0.4 |
| 150-160 | 941 | 21.6 | 1.41 (1.13-1.76) | 0.002 | 1.32 (1.06-1.65) | 0.014 |
| ≥160 | 377 | 20.1 | 1.42 (0.99-2.04) | 0.054 | 1.37 (0.96-1.97) | 0.085 |
| LDL-C (mg/dL)* | | | | | | |
| <70 | 676 | 33.7 | 1.99 (1.61-2.45) | <0.001 | 1.75 (1.42-2.16) | <0.001 |
| 70-100 | 3604 | 19.6 | 1.22 (1.07-1.40) | 0.003 | 1.18 (1.03-1.35) | 0.014 |
| 100-130 | 6051 | 14.1 | reference |  | reference |  |
| 130-160 | 1806 | 19.5 | 1.43 (1.21-1.69) | <0.001 | 1.47 (1.24-1.74) | <0.001 |
| ≥160 | 506 | 31.9 | 2.37 (1.89-2.98) | <0.001 | 2.30 (1.83-2.89) | <0.001 |

The models used Cox proportional hazards regression analyses adjusted for potential confounders. *HbA1c, SBP, and LDL-C were calculated as the mean of any values recorded between the index date and death or censor.

Model 1 adjusted for age and sex.

Model 2 included the confounders in model 1, plus pre-existing myocardial infarction, congestive heart failure, stroke, malignant neoplasm, chronic kidney disease, use of insulin, any anti-hypertensive drug, any lipid-lowering drug, and antiplatelet.
