# Supplementary material for: All-Cause Mortality in Patients with Type 2 Diabetes in Association with Achieved Hemoglobin A1c, Systolic Blood Pressure, and Low-Density Lipoprotein Cholesterol Levels
Source: PLoS One. 2014 Oct 27;9(10):e109501. doi: 10.1371/journal.pone.0109501 (PMC4210124; doi:10.1371/journal.pone.0109501)
Supplement: Table S2 — Cox proportional hazard models for all-cause mortality introducing achieved HbA1c, SBP, or LDL-C as updated, cumulative, yearly mean values. (DOCX) [file pone.0109501.s002.docx]

**Table S2. Cox proportional hazard models for all-cause mortality introducing achieved HbA1c, SBP, or LDL-C as updated, cumulative, yearly mean values**

|  | Hazard ratio (95% CI) | *P* value | Hazard ratio (95% CI) | *P* value |
| --- | --- | --- | --- | --- |
| HbA1c (%)* | Model 1 | | Model 2 | |
| <6.0 | 1.60 (1.27-2.00) | <0.001 | 1.65 (1.31-2.07) | <0.001 |
| 6.0-7.0 | 1.14 (0.97-1.33) | 0.12 | 1.21 (1.03-1.42) | 0.021 |
| 7.0-8.0 | reference |  | reference |  |
| 8.0-9.0 | 1.23 (1.05-1.45) | 0.011 | 1.20 (1.02-1.41) | 0.028 |
| 9.0-10.0 | 1.38 (1.13-1.68) | 0.001 | 1.27 (1.04-1.55) | 0.018 |
| ≥10.0 | 1.71 (1.40-2.08) | <0.001 | 1.61 (1.32-1.96) | <0.001 |
| SBP (mmHg)* | Model 3 | | Model 4 | |
| <120 | 0.97 (0.76-1.23) | 0.8 | 1.05 (0.82-1.34) | 0.7 |
| 120-130 | 1.07 (0.93-1.23) | 0.4 | 1.13 (0.98-1.30) | 0.089 |
| 130-140 | reference |  | reference |  |
| 140-150 | 1.02 (0.88-1.19) | 0.8 | 1.02 (0.87-1.19) | 0.8 |
| 150-160 | 1.15 (0.92-1.44) | 0.23 | 1.14 (0.91-1.43) | 0.27 |
| ≥160 | 1.09 (0.78-1.52) | 0.6 | 1.10 (0.79-1.54) | 0.6 |
| LDL-C (mg/dL)* | Model 5 | | Model 6 | |
| <70 | 1.78 (1.45-2.18) | <0.001 | 1.64 (1.34-2.01) | <0.001 |
| 70-100 | 1.16 (1.01-1.32) | 0.033 | 1.11 (0.97-1.27) | 0.14 |
| 100-130 | reference |  | reference |  |
| 130-160 | 1.11 (0.94-1.32) | 0.20 | 1.13 (0.96-1.34) | 0.15 |
| ≥160 | 1.82 (1.46-2.27) | <0.001 | 1.81 (1.45-2.27) | <0.001 |

The models used Cox proportional hazards regression analyses adjusted for potential confounders. *HbA1c, SBP, or LDL-C was introduced in an updated, cumulative, yearly mean value (with the last observation carried forward for missing data).

Model 1 adjusted for age, sex, mean SBP, and LDL-C.

Model 3 adjusted for age, sex, mean HbA1c, and LDL-C.

Model 5 adjusted for age, sex, mean HbA1c, and SBP.

Model 2,4,6 included the confounders in model 1,2,3, respectively, plus pre-existing myocardial infarction, congestive heart failure, stroke, malignant neoplasm, chronic kidney disease, use of insulin, any anti-hypertensive drug, any lipid-lowering drug, and antiplatelet.
