# Supplementary material for: All-Cause Mortality in Patients with Type 2 Diabetes in Association with Achieved Hemoglobin A1c, Systolic Blood Pressure, and Low-Density Lipoprotein Cholesterol Levels
Source: PLoS One. 2014 Oct 27;9(10):e109501. doi: 10.1371/journal.pone.0109501 (PMC4210124; doi:10.1371/journal.pone.0109501)
Supplement: Table S3 — Cox proportional hazard models in subgroups. (DOCX) [file pone.0109501.s003.docx]

**Table S3. Cox proportional hazard models in subgroups**

|  | Patient number | Mortality rate (per 1000 person-years) | Hazard ratio (95% CI) | *P* value | Patient number | Mortality rate (per 1000 person-years) | Hazard ratio (95% CI) | *P* value |
| --- | --- | --- | --- | --- | --- | --- | --- | --- |
| HbA1c (%)* |  | | Model 1: with insulin | |  | | Model 2: with only oral hypoglycemic agents | |
| <6.0 | 53 | 103.6 | 2.26 (1.41-3.64) | <0.001 | 391 | 35.0 | 2.09 (1.60-2.74) | <0.001 |
| 6.0-7.0 | 247 | 44.1 | 1.34 (0.94-1.92) | 0.11 | 2418 | 18.8 | 1.42 (1.18-1.70) | <0.001 |
| 7.0-8.0 | 502 | 34.6 | reference |  | 3685 | 11.8 | reference |  |
| 8.0-9.0 | 477 | 29.1 | 0.91 (0.67-1.24) | 0.6 | 2281 | 13.9 | 1.32 (1.09-1.60) | 0.004 |
| 9.0-10.0 | 339 | 26.7 | 0.97 (0.68-1.38) | 0.9 | 1069 | 14.4 | 1.55 (1.22-1.97) | <0.001 |
| ≥10.0 | 320 | 33.5 | 1.47 (1.04-2.08) | 0.031 | 861 | 18.4 | 2.35 (1.84-2.99) | <0.001 |
| SBP (mmHg)* |  | | Model 3: with baseline hypertension | |  | | Model 4: without baseline hypertension | |
| <120 | 238 | 27.9 | 1.48 (1.02-2.15) | 0.041 | 596 | 11.5 | 1.37 (0.94-1.99) | 0.10 |
| 120-130 | 1177 | 27.9 | 1.47 (1.22-1.77) | <0.001 | 2005 | 13.9 | 1.25 (1.00-1.56) | 0.050 |
| 130-140 | 2441 | 20.5 | reference |  | 2176 | 12.2 | reference |  |
| 140-150 | 1295 | 21.6 | 1.10 (0.92-1.31) | 0.30 | 897 | 13.1 | 0.96 (0.72-1.29) | 0.8 |
| 150-160 | 698 | 23.7 | 1.33 (1.04-1.70) | 0.025 | 243 | 15.2 | 1.31 (0.79-2.17) | 0.30 |
| ≥160 | 300 | 20.4 | 1.35 (0.90-2.02) | 0.15 | 77 | 19.3 | 1.37 (0.59-3.22) | 0.5 |
| LDL-C (mg/dL)* |  | | Model 5: with baseline hyperlipidemia | |  | | Model 6: without baseline hyperlipidemia | |
| <70 | 154 | 24.4 | 1.31 (0.74-2.33) | 0.4 | 522 | 35.8 | 1.87 (1.49-2.35) | <0.001 |
| 70-100 | 787 | 18.5 | 1.22 (0.88-1.70) | 0.23 | 2817 | 19.8 | 1.18 (1.02-1.37) | 0.028 |
| 100-130 | 1293 | 13.9 | reference |  | 4758 | 14.1 | reference |  |
| 130-160 | 487 | 15.2 | 1.18 (0.79-1.75) | 0.4 | 1319 | 20.8 | 1.55 (1.29-1.86) | <0.001 |
| ≥160 | 166 | 30.8 | 1.78 (1.13-2.78) | 0.012 | 340 | 32.4 | 2.50 (1.91-3.27) | <0.001 |

The models used Cox proportional hazards regression analyses adjusted for potential confounders. *HbA1c, SBP, and LDL-C were calculated as the mean of any values recorded between the index date and death or censor.

Model 1,2 adjusted for age, sex, mean SBP, LDL-C, pre-existing myocardial infarction, congestive heart failure, stroke, malignant neoplasm, chronic kidney disease, use of insulin, any anti-hypertensive drug, any lipid-lowering drug, and antiplatelet.

Model 3,4 included the confounders in model 1,2 minus mean SBP, but plus mean HbA1c.

Model 5,6 included the confounders in model 1,2 minus mean LDL-C, but plus mean HbA1c.
